# Red and Infrared Low-Level Laser Therapy Prior to Injury with or without Administration after Injury Modulate Oxidative Stress during the Muscle Repair Process

**DOI:** 10.1371/journal.pone.0153618

**Published:** 2016-04-15

**Authors:** Beatriz Guimarães Ribeiro, Agnelo Neves Alves, Lucas Andreo Dias dos Santos, Tatiane Matarazzo Cantero, Kristianne Porta Santos Fernandes, Danielle da Silva Dias, Nathalia Bernardes, Kátia De Angelis, Raquel Agnelli Mesquita-Ferrari

**Affiliations:** 1 Rehabilitation Department, Universidade Nove de Julho (UNINOVE), São Paulo, SP, Brazil; 2 Biophotonics Department, Universidade Nove de Julho (UNINOVE), São Paulo, SP, Brazil; 3 Hypertension Unit, Heart Institute (InCor), School of Medicine, University of São Paulo, São Paulo, SP, Brazil; 4 Medicine Department, Universidade Nove de Julho (UNINOVE), São Paulo, SP, Brazil; Alexandria University, EGYPT

## Abstract

**Introduction:**

Muscle injury is common among athletes and amateur practitioners of sports. Following an injury, the production of reactive oxygen species (ROS) occurs, which can harm healthy muscle fibers (secondary damage) and delay the repair process. Low-level laser therapy (LLLT) administered prior to or following an injury has demonstrated positive and protective effects on muscle repair, but the combination of both administration times together has not been clarified.

**Aim:**

To evaluate the effect of LLLT (660 nm and 780 nm, 10 J/cm², 40 mW, 3.2 J) prior to injury with or without the administration after injury on oxidative stress during the muscle repair process.

**Methods:**

Wistar rats were divided into following groups: control; muscle injury alone; LLLT 660 nm + injury; LLLT 780 nm + injury; LLLT 660 nm before and after injury; and LLLT 780 nm before and after injury. The rats were euthanized on days 1, 3 and 7 following cryoinjury of the tibialis anterior (TA) muscle, which was then removed for analysis.

**Results:**

Lipid peroxidation decreased in the 660+injury group after one day. Moreover, red and infrared LLLT employed at both administration times induced a decrease in lipid peroxidation after seven days. CAT activity was altered by LLLT in all periods evaluated, with a decrease after one day in the 780+injury+780 group and after seven days in the 780+injury group as well as an increase in the 780+injury and 780+injury+780 groups after three days. Furthermore, increases in GPx and SOD activity were found after seven days in the 780+injury+780 group.

**Conclusion:**

The administration of red and infrared laser therapy at different times positively modulates the activity of antioxidant enzymes and reduces stress markers during the muscle repair process.

## Introduction

Muscle injury is common in athletes and amateur practitioners of sports and reduces the performance of them [1,2]. After an injury, the muscle repair process begins and is divided into interdependent phases: degeneration and inflammation, regeneration, fibrosis/scar formation and remodeling [3].

During the acute phase, the release of reactive oxygen species (ROS) occurs, which are products of the mitochondrial oxidative metabolism of inflammatory cells, endothelial cells and muscle cells [4]. The production of ROS at adequate levels in combination with growth factors and cytokines is important to the muscle repair process due to the redirection of myogenic precursor cells (satellite cells) to the injury site [5]. However, high levels of ROS for a long period of time in the injured area can cause oxidative harm (secondary damage) by directly reaching vital cell constituents, such as lipids, proteins and DNA, in addition to interfering negatively in the differentiation of muscle cells [6].

ROS during the muscle repair process depend on the capacity of cellular antioxidant enzymes, such as superoxide dismutase (SOD), catalase (CAT) and glutathione peroxidase (GPx), to control their harmful effects [5]. These enzymes allow a delicate state of balance between ROS levels produced during metabolism and eliminated by the antioxidant system, denominated the cellular redox state [7]. An imbalance in the redox state favoring ROS is termed oxidative stress, which is determined by an increase in protein oxidation and lipid peroxidation [8].

Photobiomodulation employs monochromatic light in the optical region of red and infrared lasers to treat various tissues in a non-destructive and non-thermal fashion [9]. Treatment is based on the ability of light to alter the cell metabolism, particularly as a result of being absorbed by mitochondria and cytochrome C oxidase [10]. Low-level laser therapy (LLLT) applied prior to [11] or following an injury [12] has demonstrated positive and protective effects on muscle repair, including the modulation of the inflammatory process [11,13], angiogenesis [11,13,14], collagen remodeling [11,13,14,15] as well as the formation of immature muscle fibers [11,13]. However, the combination of the both administration times together has not been clarified.

Thus, the aim of the present study was to investigate the effects of LLLT administered prior to muscle injury with or without administration after injury on oxidative stress.

## Materials and Methods

This study received approval from the local ethics committee (process number AN25/2014) and all experiments were performed in accordance with the guidelines of the Brazilian National Council for the Control of Animal Experimentation. Wistar rats (n = 80) weighing 250 ± 15 g were divided into five groups: control group (n = 5); injury group alone (n = 15); LLLT 660 nm prior to injury (n = 15); LLLT 780 nm prior to injury (n = 15); LLLT 660 nm applied before and after injury (n = 15); and LLLT 780 nm administered before and after injury (n = 15). The control group was euthanized on the first day after the onset of the experiment. The other groups were euthanized on days 1, 3 and 7 after the induction of injury.

### Low-level laser irradiation therapy

LLLT was performed in point mode directly on the skin overlying the tibialis anterior (TA) muscle using a Twin Laser ® (MM Optics, São Carlos—SP, Brazil) with wavelengths of 660 nm (active medium: aluminum-gallium-indium-phosphide [AlGaInP]) and 780 nm (active medium: aluminum-gallium-arsenide [AlGaAs]). The parameters were based on studies described by Ribeiro et al. [11] and Alves et al. [13] (Table 1). LLLT was only administered once immediately prior to the induction of injury in the LLLT + injury groups. When LLLT was administered both before and after injury, irradiation was performed once immediately prior to injury and two hours following injury, along with treatments every day until euthanasia.

**Table 1:** Low-level laser therapy parameters.

Active medium	Wavelength	Beam area	Power output	Power density	Energy density	Energy per point	Total points	Time per point	Total time	Total energy
AlGaInP	660 nm	0.04 cm^2^	40 mW	1 W/cm^2^	10 J/cm^2^	0.4 J	8	10 seconds	80 seconds	3.2 J
AlGaAs	780 nm									

### Cryoinjury procedure

Cryoinjury consisted of two applications of a metal rod (3 mm in diameter) cooled in liquid nitrogen to the ventral surface of the TA muscle [11,13,14,15]. At the end of the experimental protocol, the animals were euthanized with an overdose of anesthesia and the TA muscles were removed for analysis.

### Oxidative stress

#### Oxidative stress profile on skeletal muscle tissue

The skeletal muscle tissue was macerated in liquid nitrogen. Five mL 150 mM of KCl and 20 nM of sodium phosphate buffer (pH 7.4) were added per 1 g of tissue. The homogenate was centrifuged at 600 g for 10 min at -26°C [16].

#### Chemiluminescence

The lipid peroxidation membrane was evaluated by chemiluminescence (CL). The CL assay was conducted with a Tri-Carb 2800 TR Liquid Scintillation Analyzer (PerkinElmer, USA) in the out-of-coincidence mode at room temperature (25°C to 27°C). The supernatants were diluted in 140 mM of KCl and 20 mM of sodium phosphate buffer, pH 7.4, and placed into glass tubes, which were placed in scintillation vials. Three mM of tert-butylhydroperoxide were added and CL was determined up to the maximum level of emission [16,17,18].

#### Protein oxidation

Protein oxidation was measured using a reaction of protein carbonyl groups with 2,4-dinitrofenylhydrazyne to form 2,4-dinitrophenylhydrazone, which can be measured spectrophotometrically. The reaction product was measured at 360 nm. The results were expressed as nanomolars of 2,4-dinitrofenylhydrazyne per milligram of protein [19,20].

#### Antioxidant enzyme activity

The quantification of SOD activity (expressed as U/mg of protein) was based on the inhibition of the reaction between O_2_ and pyrogallol. CAT activity was determined by measuring the decrease in H_2_O_2_ absorbance at 240 nm and expressed as μmol H_2_O_2_ reduced/min/mg of protein. GPx activity was expressed as nmol peroxide/hydroperoxide reduced/min/mg of protein and was based on the consumption of NADPH at 480 nm [16].

### Statistical analysis

The Kolmogorov-Smirnov test ​was used to determine the distribution (normal or non-normal) of the variables and demonstrated parametric data, which were expressed as mean and standard error of the mean (SEM). ANOVA was used for the comparisons between groups, followed by Tukey’s post hoc test. Results were considered statistically significant when the p-value was ≤ 0.05. The data were analyzed using the BioStat 5.0 software program.

## Results

### Lipid peroxidation

**Fig 1:**
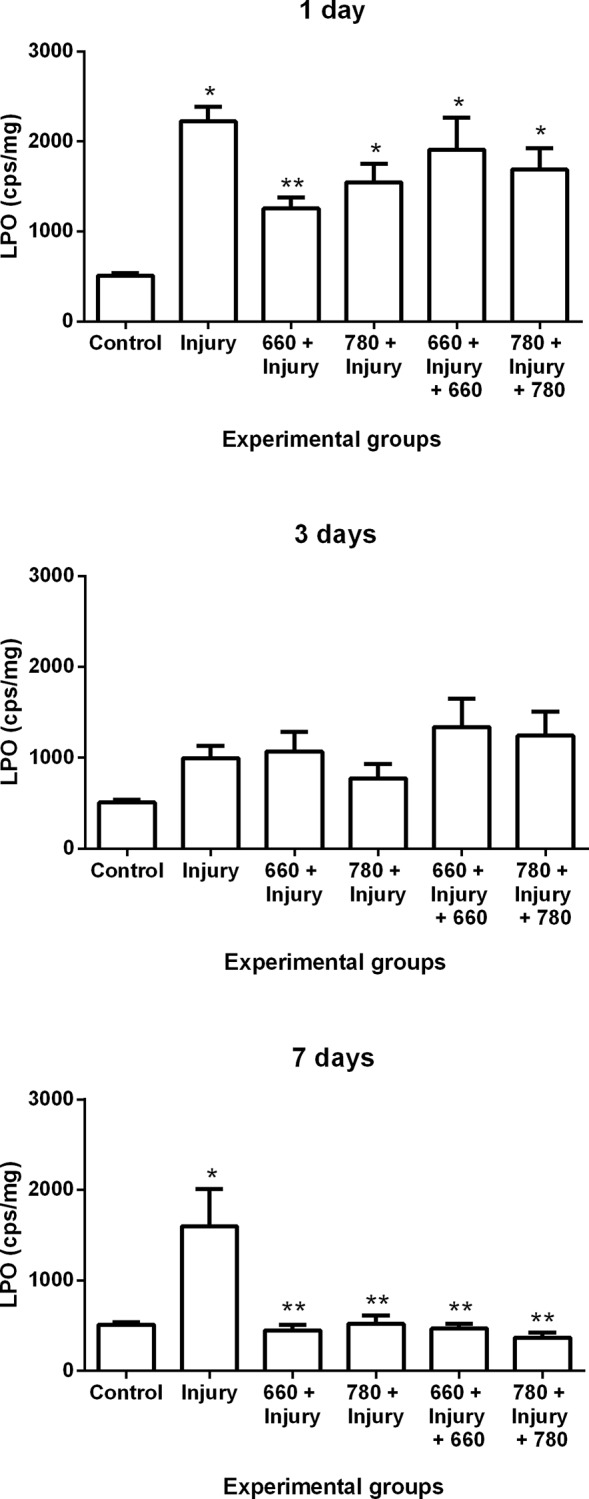
Lipid peroxidation in different experimental groups; values expressed as mean and SEM. ANOVA/Tukey’s test; *p ≤ 0.05 compared to control group; **p ≤ 0.05 compared to injury group.

Lipid peroxidation (LPO) increased in the injury, 780 + injury, 660 + injury + 660 and 780 + injury + 780 groups in comparison to control group and decreased in the 660 + injury group in comparison to the injury group after one day. Furthermore, there was an increase in LPO in the injury group in comparison to control group and a decrease in LPO in all irradiation groups in comparison to the injury group after seven days (Fig 1).

### Protein oxidation

**Fig 2:**
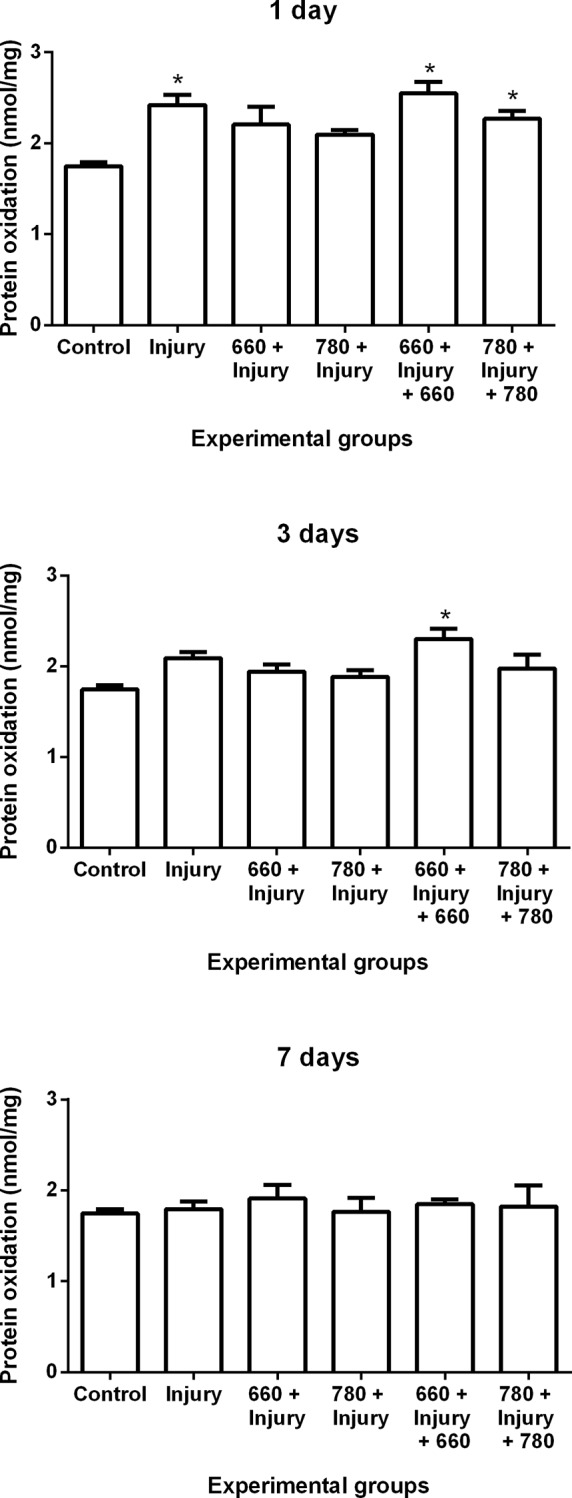
Protein oxidation in different experimental groups; values expressed as mean and SEM. ANOVA/Tukey’s test; *p ≤ 0.05 compared to control group

Protein oxidation increased in the injury, 660 + injury + 660 and 780 + injury + 780 groups in comparison to the control group after one day. At three days, there was an increase of protein oxidation in the 660 + injury + 660 group in comparison to the control group. No significant differences were found among groups after seven days (Fig 2).

### Antioxidant enzyme activity

#### Superoxide dismutase (SOD) activity

**Fig 3:**
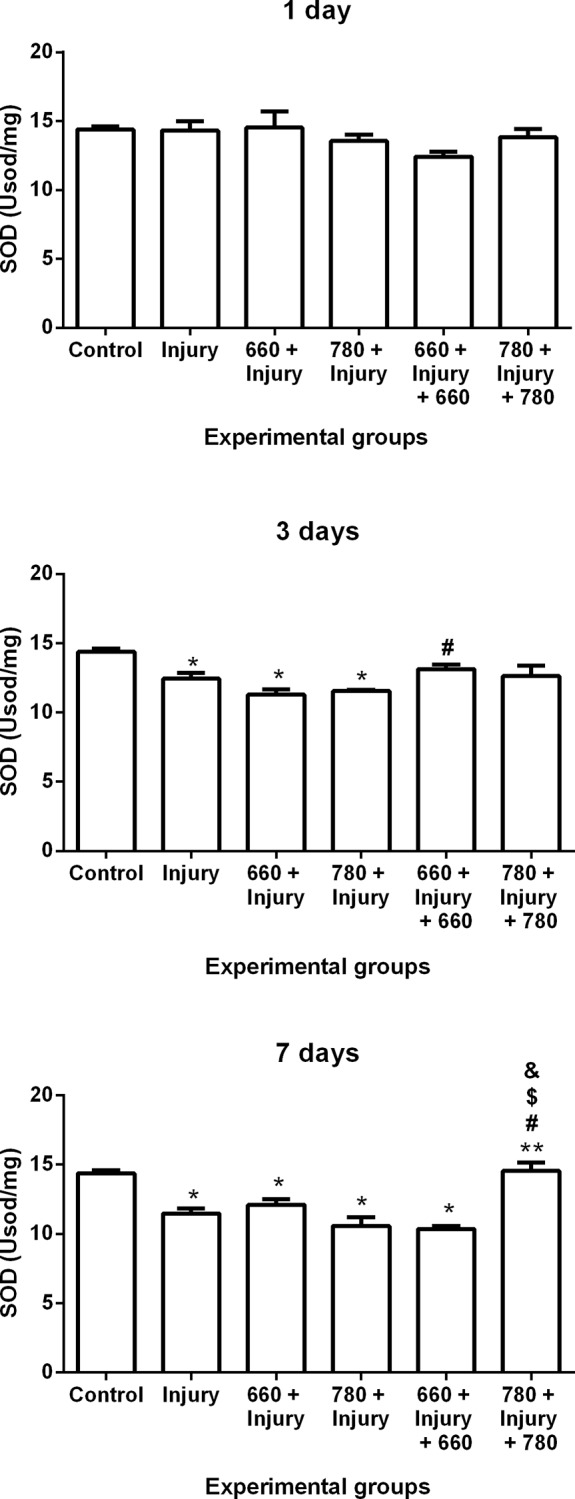
SOD activity in different experimental groups; values expressed as mean and SEM; ANOVA/Tukey’s test; *p ≤ 0.05 compared to control group; **p ≤ 0.05 compared to injury group; #p ≤ 0.05 compared to 660 + injury group; $p ≤ 0.05 compared to 780 + injury group; &p ≤ 0.05 compared to 660 + injury + 660 group.

SOD activity decreased in the injury, 660 + injury and 780 + injury groups in comparison to the control group and increased in the 660 + injury + 660 group in comparison to 660 + injury group after three days. Moreover, SOD activity decreased in the injury, 660 + injury, 780 + injury and 660 + injury + 660 groups in comparison to the control group. SOD activity increased in the 780 + injury + 780 group in comparison to the injury, 660 + injury, 780 + injury and 660 + injury + 660 groups after seven days (Fig 3).

#### Catalase (CAT) activity

**Fig 4:**
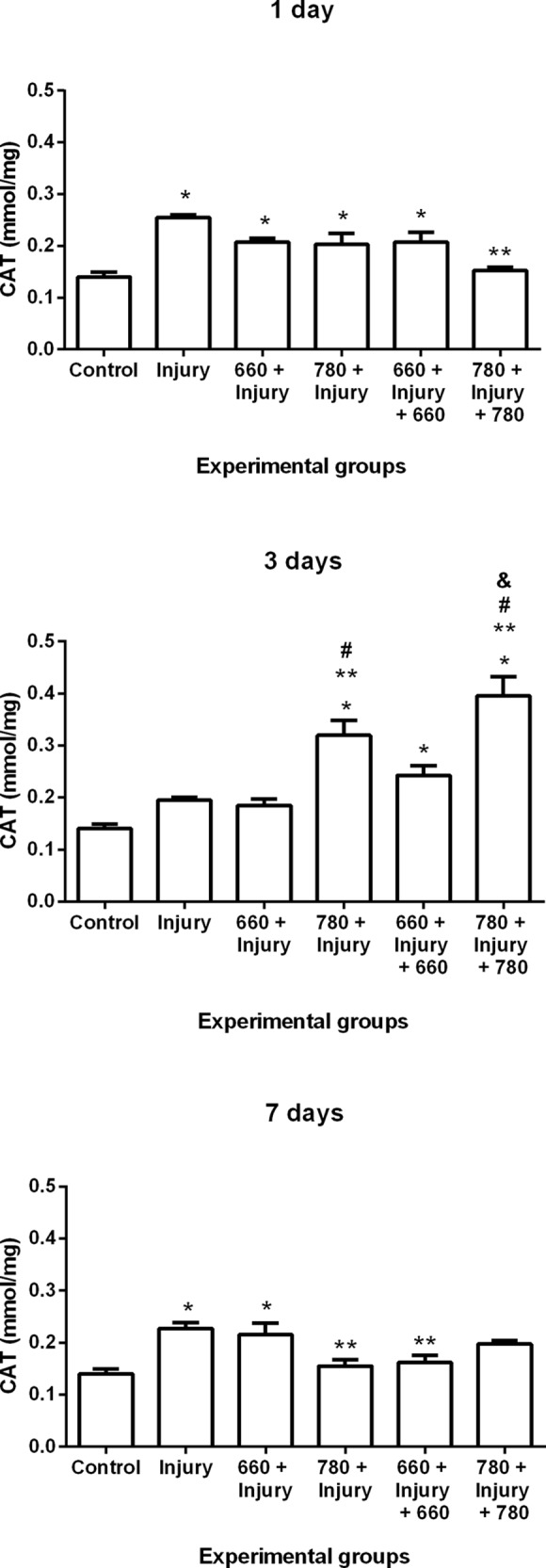
CAT activity in different experimental groups; values expressed as mean and SEM; ANOVA/Tukey’s test; *p ≤ 0.05 compared to control group; **p ≤ 0.05 compared to injury group; #p ≤ 0.05 compared to 660 + injury group; &p ≤ 0.05 compared to 660 + injury + 660 group.

The results demonstrated an increase in CAT activity in the injury, 660 + injury, 780 + injury and 660 + injury + 660 groups in comparison to the control group after one day. Moreover, a reduction in CAT activity was found after one day in the 780 + injury + 780 group in comparison to the injury group. CAT activity increased in the 780 + injury, 660 + injury + 660 and 780 + injury + 780 groups in comparison to the control group as well as in the 780 + injury and 780 + injury + 780 groups in comparison the injury group after three days. An increase in activity was also found in the 780 + injury and 780 + injury + 780 groups in comparison to the 660 + injury group and in the 780 + injury + 780 group in comparison to 660 + injury + 660 group after three days. After seven days, an increase in CAT activity was found in the injury and 660 + injury groups in comparison to the control group and a decrease was found in the 780 + injury and 660 + injury + 660 groups in comparison to injury group (Fig 4).

#### Glutathione peroxidase activity

**Fig 5:**
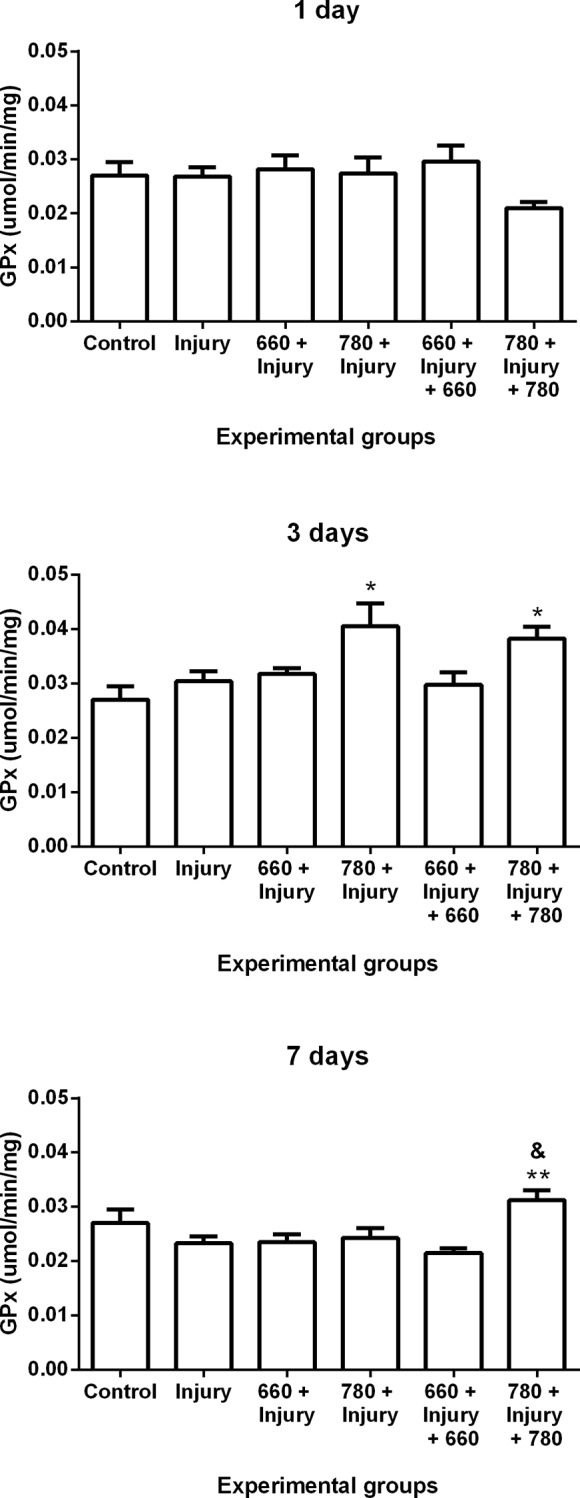
GPx activity in different experimental groups; values expressed as mean and SEM; ANOVA/Tukey’s test; *p ≤ 0.05 compared to control group; **p ≤ 0.05 compared to injury group; &p ≤ 0.05 compared to 660 + injury + 660 group.

GPX activity increased in the 780 + injury and 780 + injury + 780 groups in comparison to control group after three days and in the 780 + injury + 780 group in comparison to the injury group after seven days. The groups irradiated with red LLLT (660 nm) demonstrated no significant differences in comparison to the other groups (Fig 5).

## Discussion and Conclusion

ROS participate in the cascade of muscle regeneration events, but elevated ROS levels for long periods can lead to oxidative damage as well as increased inflammation and therefore affect the differentiation of muscle cells [5]. Thus, researchers have sought therapeutic strategies to minimize the harmful production of ROS to allow adequate muscle repair.

LLLT has demonstrated a protective effect on muscle tissue, as it modulates mitochondrial activity for the synthesis of adenosine triphosphate and ROS [21,22,23]. However, there are no reports in the literature on the effect of laser administration prior to injury combined or not with post-injury irradiation on oxidative stress. To the best of our knowledge, this is the first study to compare the effects of different LLLT administration times on muscle injury. The findings suggest that the administration of LLLT at different times positively modulates oxidative stress and the effects were more accentuated in the groups that received infrared LLLT.

Lipid peroxidation is a process that can be initiated by ROS and can block cell proliferation as well as induce apoptosis and necrosis, thereby increasing tissue damage [24]. There is evidence that the increase in ROS is associated with increased muscle damage and the formation of fibrosis in skeletal muscles [25]. Studies using LLLT prior to injury [11] or after injury [13,26,27] have shown reduced muscle damage and collagen deposition during the repair process.

In the present study, irradiation with red LLLT prior to muscle injury induced a decrease in the lipid peroxidation after one day, which is in agreement with data reported in the literature. Luo et al. [25] used red laser (635 nm and 7 mW) on rat gastrocnemius muscles during the repair process following contusion and found a decrease in lipid peroxidation (malondialdehyde) after one day. Liu et al. [28] used a red laser (632.8 nm, 14 mW and 43 J/cm²) on the gastrocnemius muscle of rats in the repair process following eccentric exercise and demonstrated an increase in lipid peroxidation (malondialdehyde) after one and two days.

Moreover, red and infrared LLLT at both administration times induced a decrease in lipid peroxidation after seven days, which is the same result found in studies that only evaluated the effect of infrared laser following a muscle injury. Using LLLT (808 nm, 30 mW, 180 J/cm^2^ and 1.4 J) following cryoinjury to the TA muscle, Assis et al. [29] demonstrated a decrease in lipid peroxidation (TBARS) after four days. Silveira et al. [30] also demonstrated that treatment with pulsed LLLT (904 nm, 40 mW, pulse 60 ns, 9500 Hz, 5 J/cm^2^ and 2.5 J) following trauma to the gastrocnemius muscle caused a decrease in lipid peroxidation (TBARS) after five days.

Furthermore, lipid peroxidation is inhibited by antioxidant enzymes, such as SOD, CAT and GPx, as these enzymes reduce ROS levels [8]. The analysis of antioxidant enzymes in the present investigation demonstrated that infrared laser prior to injury can lead to a decrease in CAT activity after seven days. Prior infrared laser with or without the post-injury treatment had a more favorable modulatory effect on CAT, as it induced an increase in its activity after three days. Moreover, prior infrared laser combined with post-irradiation was more favorable to the promotion of the down-regulation of CAT activity after one day and the promotion of the up-regulation of GPx and SOD activity after seven days in comparison to the injury group. Enzyme activity was similar to baseline activity (control group), especially in the groups that received both prior and post infrared and red irradiation.

Urishi et al. [31] proposes that antioxidant levels are determinants of the regenerative capacity of muscle stem cells. Thus, the up-regulation of antioxidant enzymes activity found in the present study could partially explain the findings reported by Ribeiro et al. [11], who used infrared LLLT prior to injury and Alves et al. [13] who used infrared LLLT after injury and demonstrated an increase in the numbers of immature muscle fibers as well as a decrease in muscle damage after seven days.

Other studies using red and infrared LLLT following injury and the same experimental model (cryoinjury to the TA muscle) demonstrated positive effects during muscle repair, but nothing was described regarding oxidative stress. Both red and infrared LLLT have positive effects on the modulation of the inflammatory process, inducing a reduction in inflammatory cytokines [32,33] and myonecrosis [13,15]. Moreover, the induction in extracellular matrix remodeling leads to the increased deposition of collagen types I, III and IV (red LLLT) [14,15] as well as an improvement in their distribution and organization (infrared LLLT) [13,29]. Infrared LLLT causes an increase in immature muscle fibers [13] and the modulation of myogenic regulatory factors [29,34,35]. The studies cited demonstrate the importance of laser irradiation on the muscle repair process independently of the wavelength used. However, these studies only used LLLT after injury

The present investigation aimed to evaluate the effect of the prior administration effect of LLLT combined or not with post-treatment LLLT to allow an understanding of the response of the irradiated muscle tissue when it is still healthy and the influence of these effects on the muscle repair process after an acute injury. This is especially important to the establishment of therapeutic strategies in patients with the imminent risk of muscle injuries, such as athletes.

In conclusion, red and infrared laser therapy proved effective to the positive modulation of antioxidant enzyme activity and the reduction in stress markers during the muscle repair process, independently of the administration time, whereas the effects of infrared LLLT were more pronounced with regard to modulating antioxidant enzymes.
